# GlucoNeo Check in the community: feasibility of photoplethysmography-based non-invasive glucose screening

**DOI:** 10.3389/ijph.2026.1609599

**Published:** 2026-07-22

**Authors:** Mahendro Prasetyo Kusumo, Slamet Riyadi, Karisma Trinanda Putra, Deasy Ardiany

**Affiliations:** 1 Master of Hospital Administration, Faculty of Medicine and Health Sciences, Universitas Muhammadiyah Yogyakarta, Yogyakarta, Indonesia; 2 Information Technology, Faculty of Engineering, Universitas Muhammadiyah Yogyakarta, Yogyakarta, Indonesia; 3 Electrical Technology, Faculty of Engineering, Universitas Muhammadiyah Yogyakarta, Yogyakarta, Indonesia; 4 Division of Endocrinology and Metabolism, Department of Internal Medicine, Faculty of Medicine, Universitas Airlangga, Dr. Soetomo General Academic Hospital, Surabaya, Indonesia

**Keywords:** artificial intelligence (AI), community diabetes prevention, non-invasive glucose monitoring, PPG signals, wearable devices

## Abstract

**Objective:**

This study aims to determine accuracy of GlucoNeo Check, PPG-Based Non-Invasive Glucose Screening Device in interpreting patients’ blood glucose levels and to understand participants’ perspectives on their experiences.

**Method:**

This study uses a mixed-methods with a cross-sectional diagnostic accuracy design to obtain a comprehensive overview through Clarke Error Grid Analysis using 660 participants with 3300 data sets, while incorporating qualitative components to assess feasibility and acceptance, using interviews and FGDs from 31 respondents, and analyzed thematically to understand user experience perspectives when using the device.

**Results:**

GlucoNeo Check performance had a Mean Absolute Percentage Error of 3.6% ± 0.621 and classification accuracy of 89.2% ± 0.0515. Most data fell within Zone A, with a small amount in Zone B and no data in Zones C and D. Thematic analysis identified 4 themes: 1) device quality and practicality, 2) comfort of use, 3) facilitating technology, and 4) inclusive interface design.

**Conclusion:**

GlucoNeo Check is accurate, innovative, and well-received tool for supporting community-based diabetes prevention but requires further validation to be adopted as a standard clinical monitoring tool.

## Introduction

The global prevalence of diabetes continues to show a significant upward trend. Based on data from Riskesdas 2018, the prevalence of diabetes in Indonesia reached 10.9%, with over 73.7% of sufferers unaware that they have the disease [1]. Data shows that diabetes also affects over 10.5% of the adult population, with an estimated increase in incidence to 11.3% by 2030 and 12.2% by 2045 [2, 3]. Yogyakarta has the second-highest prevalence of diabetes in Indonesia and is among the non-communicable diseases with the highest incidence rates, along with hypertension and cancer [3]. The continuous increase in almost all countries around the world makes it necessary to constantly monitor projections and estimates of diabetes sufferers in order to prevent and prepare appropriate and accurate treatment for people with diabetes [1]. Most of these patients had never undergone routine checkups, indicating a continued need to raise awareness about the importance of regular blood glucose testing to maintain overall health [4].

Routine blood glucose testing is an important component and supportive step in long-term management of Diabetes, because high blood glucose levels have the potential to cause further complications [2, 5]. Routine monitoring also provides sufficient time for patients to take early action, to delay the incidence and progression of diabetes-related complications or even prevent diabetes from occurring altogether [6]. Invasive finger pricks glucometers, as standard tool for blood glucose measurement, require painful blood sampling that reduce patient comfort with potential of infection, creating barrier to repeated testing, decreasing adherence to disease management [7–9]. Furthermore, for elderly patients or those with a fear of needles, it can cause symptoms of anxiety, making invasive blood glucose monitoring a significant challenge [10].

In response to these challenges, minimally-invasive technologies such as Continuous Glucose Monitoring (CGM) have emerged, utilizing microneedles to monitor glucose in the interstitial fluid with high clinical correlation (R = 0.90–0.95). However, these systems still require skin penetration, involve a physiological lag of 5–15 min compared to blood glucose, and necessitate periodic sensor replacements, which may still pose a burden for some users. Development of non-invasive glucose monitoring is therefore considered the “Holy Grail” in diabetes monitoring technology because it has the potential to improve the quality of life while also ideal for reducing discomfort, the risk of infection, and improving convenience, as well as providing information about treatment options such as insulin initiation and titration [11, 12]. Consequently, non-invasive blood glucose monitoring is becoming a more convenient option [13]. Data from the UK estimates that with improved diabetes management, spending can be saved by up to 430 million Pounds in the first five years [1].

To address these issues, this research focuses on developing the GlucoNeo Check tool, which uses the MAX30102 optical sensor to capture raw PPG signals. These approaches also contribute to Community-based diabetes management, since it has been a strategy for controlling diabetes incidence worldwide with great potential to improve the quality of life for patients and can reduce the cost of diabetes care [14, 15]. The PPG signal obtained shows a periodic oscillation pattern with varying wave amplitude and morphology characteristics between subjects, which is an important indication that the PPG signal contains latent physiological information relevant to blood glucose concentration [16, 17, 18]. Machine learning and Artificial Intelligence also have great potential to improve the utility and capabilities of PPG-based blood glucose measurement tools [19]. In this research, a Convolutional Neural Network (CNN) model was applied to classify blood glucose levels to improve the system’s accuracy and predictive capabilities [20]. This study aims to determine the accuracy of GlucoNeo Check in interpreting patients’ blood glucose levels and to understand participants’ perspectives on their experience in using GlucoNeo Check.

## Methods

This study uses a mixed methods design with purposive sampling because evaluating healthcare devices is not sufficient based solely on technical performance; it also requires evidence of feasibility and user acceptance [21]. The study employs a cross sectional diagnostic accuracy design to obtain a comprehensive overview through Clarke Error Grid Analysis and Bland Altman Plots to evaluate the clinical agreement and concordance between the measurement methods. The qualitative component assesses the feasibility and user acceptance of GlucoNeo Check through thematic analysis. Purposive sampling allows for a systematic and controlled evaluation. Purposive sampling is used to make the sample more relevant to the research objectives, thereby enhancing the study’s strength, data credibility, and results [22]. Qualitative approach will be used with a case study design to provide in depth insights into user experiences with the tool and to understand the accessibility of GlucoNeo Check as a diabetes prevention medium within the community. Conversely, a device that is easy to use but technically unstable will also not last. Therefore, qualitative findings serve as contextual evidence explaining why and how devices are acceptable, including implementation barriers not captured by quantitative metrics.

This study was conducted at four Primary Health Centers in Sleman Regency, Special Region of Yogyakarta Province, and one Hospital in Surabaya from November 2024 to November 2025 as Yogyakarta has the second highest prevalence of diabetes in Indonesia [3]. These locations were chosen to vary the data samples obtained to adapt to different conditions and to identify general patterns across all locations [23]. Variations will prevent the data from becoming non identically distributed, caused by a lack of variation in the sources, patient demographics, and data collection processes [24]. The sample size was determined by assuming margin of error of 5% and alpha error of 0.05, resulting in a minimum sample size of 384. However, this study involved 660 respondents from 5 different locations, consisting of 231 men and 429 women, aged 20–69 years (Table 1). The age distribution was as follows: 20–40 years (36 people), 41–60 years (316 people), and >60 years (308 people). Total data collected was 3300 data points, aiming to represent the accuracy of the device. This oversampling technique, which collected to account for diversity in age, gender and blood glucose level, was performed to prevent misclassification, improve performance, and balance the dataset [25–27]. The inclusion criteria are: 1) age >18 years old; 2) agrees to participate and sign informed consent; 3) able to do blood glucose test using finger pricks glucometer, while exclusion criteria.

**TABLE 1:** Participants characteristics (Yogyakarta and Surabaya, Indonesia. 2024–2025).

Characteristics	N	%
Gender		
Male	231	35
Female	429	65
Age		
20–40	36	5.5
41–60	316	47.9
>60	308	46.6
Blood glucose level		
<70 mg/dL	7	1.01
70–140 mg/dL	368	55.8
>140 mg/dL	285	43.19

Blood glucose levels were measured using dua methods: first, with a conventional finger prick glucometer, and second, with a GlucoNeo Check. While laboratory plasma glucose is the clinical gold standard, the conventional finger prick glucometer was selected as the reference standard because it represents the most feasible and ethical standard of care for mass community screening in primary healthcare settings [28]. The median of 5 consecutive measurements of GlucoNeo Check was used for data analysis to reduce motion artifacts and then compared to the readings from the finger prick glucometer. Readings were taken with intervals of 10–20 s between reading, and the PPG recording duration per trial was 15–30 s. Data splitting was performed explicitly at the subject level to prevent data leakage between the training and testing sets, ensuring that no data from the same participant appeared in both the training and testing sets simultaneously [29]. Quantitative performance was further evaluated using diagnostic metrics including sensitivity, specificity, positive predictive value (PPV), and negative predictive value (NPV) to provide a detailed assessment of classification accuracy across different glycemic states.

**FIGURE 1:**
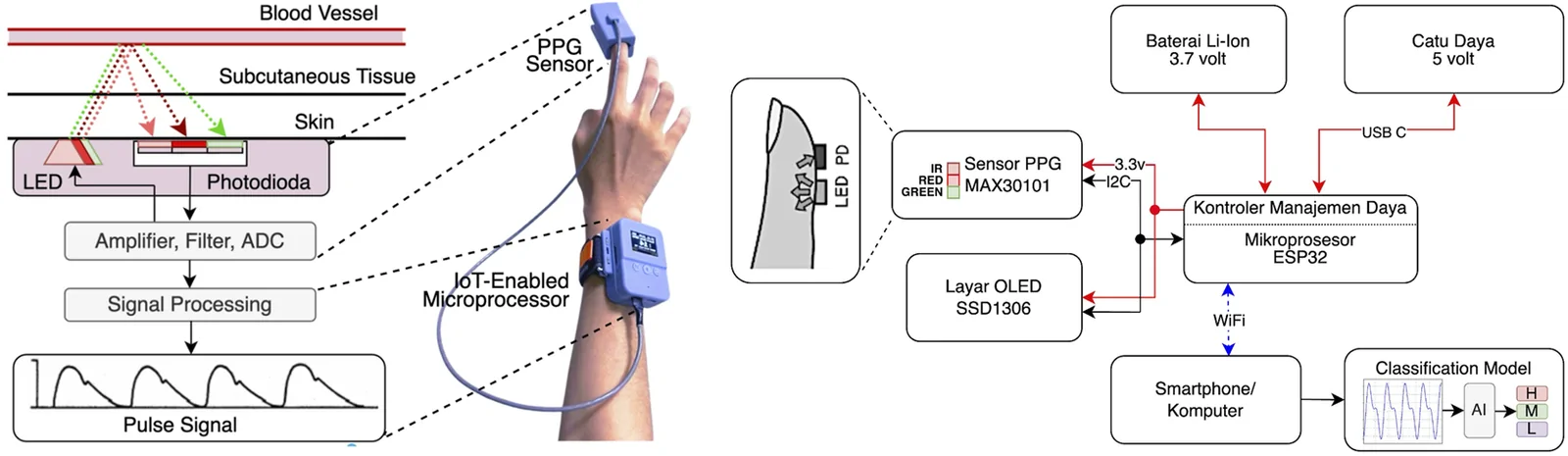
Photoplethysmography measurements collection processes (data collected can be accessed from https://www.iotmu.net) (Yogyakarta and Surabaya, Indonesia. 2024–2025).

The GlucoNeo Check utilizes a MAX30102 optical sensor to capture multi-channel PPG signals by recording volumetric changes in blood capillaries through variations in reflected light (Figure 1). To ensure transparency and facilitate future replication, the architectural framework of the Multi Task DCNN. During the pre modeling process, the raw PPG signal undergoes standardized preprocessing steps to improve data quality, including baseline drift elimination, low and high frequency noise damping, and signal normalization to reduce inter individual variability. These workflows were consistently applied across all five data collection sites to ensure reproducibility. The resulting CNN model is capable of recognizing spatiotemporal patterns within the PPG signals to predict blood glucose levels with high accuracy.

This study involving 31 qualitative respondents consisting of 20 patients, 8 healthcare professionals, and 3 experts. Data saturation was reached when no additional subthemes were identified and the data began to repeat, indicating that an adequate sample size had been achieved [30]. The respondents were part of participated participants in the quantitative data collection to gain in depth understanding of user experience in using GLucoNeo Check. Qualitative data were collected through Focus Group Discussions, followed by structured interviews that were recorded, and then summarized by the researcher. Member checking was conducted on the interview results to ensure the accuracy of the interpretation [31]. To ensure analytical rigor, the transcripts were analyzed using thematic analysis through an iterative process of open and axial coding. Two researchers independently conducted the coding process to identify recurring patterns and emerging themes, followed by peer debriefing and consensus meetings to resolve any discrepancies in categorization, thereby ensuring inter-rater reliability and the overall credibility of the findings [32, 33]. Validation was further strengthened through source triangulation, comparing user experiences, healthcare professionals’ perspectives, and cross-disciplinary expert assessments, alongside method triangulation involving focus group discussions, interviews, and document reviews. This comprehensive approach ensured that the emerging problem patterns showed strong thematic consistency. Furthermore, an audit trail was maintained throughout the research process to ensure data consistency, accuracy, and thematic transparency [32].

## Results

### Quantitative results

#### Participants characteristics

Accuracy measurement for the GlucoNeo Check involved 660 respondents from 5 different locations. Each respondent was measured once using a conventional finger prick glucometer and then remeasured using the GlucoNeo Check. The median value from 5 GlucoNeo Check readings was then taken for analysis.

#### Preprocessing and spatiotemporal extraction feature

GlucoNeo Check employs a Photoplethysmography (PPG)-based optical sensor to monitor peripheral blood volume in real-time via Red, Green, and Infrared channels. This sensor interfaces with an IoMT-based microcontroller for wireless data transmission to a central server. The PPG signal, indicative of cardiovascular activity, undergoes preprocessing to remove baseline drift, reduce noise, and normalize the signal, enhancing data quality and stability. A Deep Convolutional Neural Network (CNN) is utilized for feature extraction, and a (Bi) Long Short-Term Memory (LSTM) model captures temporal dependencies, offering insights into the spatiotemporal dynamics of the PPG signal that conventional methods lack.

#### Multi-task Deep Convolutional Neural Network (DCNN)

**FIGURE 2:**
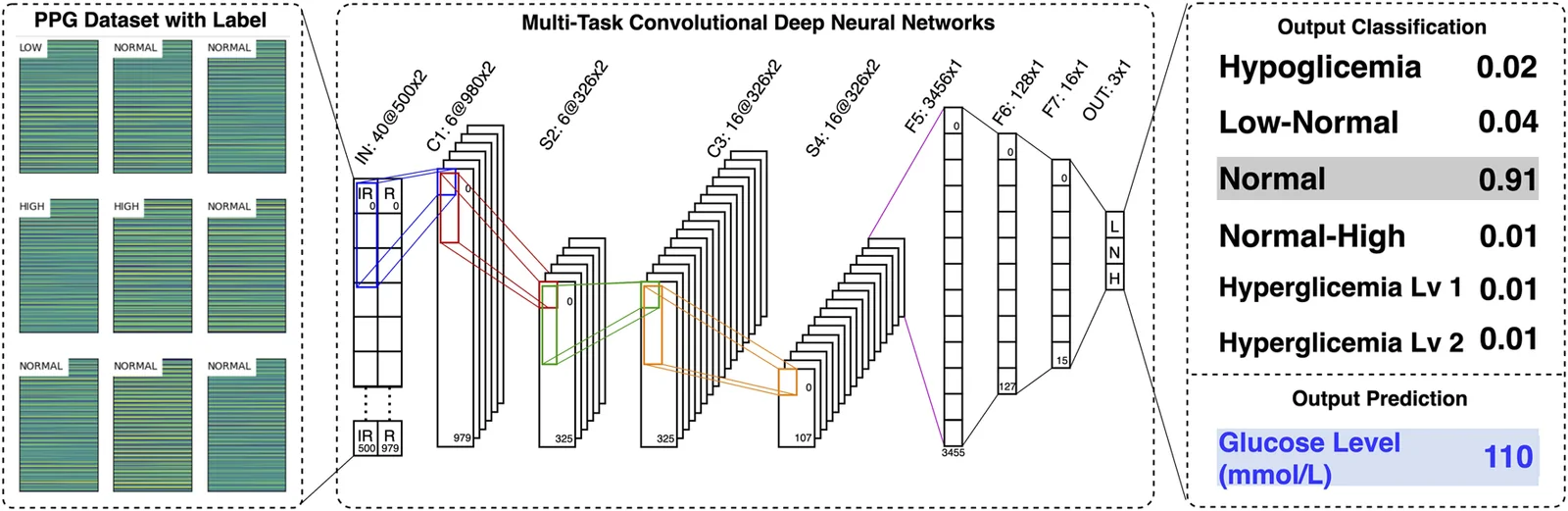
The functional architecture of the Deep Convolutional Neural Network (Yogyakarta and Surabaya, Indonesia. 2024–2025).

The proposed Multi-Task DCNN model is trained under a multi-objective learning scheme to simultaneously generate two main outputs: glycemic status classification and continuous blood glucose level regression (mg/dL) (Figure 2). This approach allows for the utilization of richer and more informative shared feature representations, thereby improving learning efficiency and overall model performance.

#### Results of glycemic status classification

Performance evaluation showed that GlucoNeo Check achieved an average accuracy of 89.2% ± 0.0515, indicating a high level of prediction consistency. Feature extraction is performed using DCNN, and then the temporal patterns are learned with (Bi) LSTM. The model is arranged in a multi-task scheme, thus producing two outputs simultaneously:

- Glycemic Status Classification
  - Hypoglycemia
  - Low-Normal (Fasting Zone)
  - Normal (Postprandial Safe)
  - Early Postprandial
  - Hyperglycemia Level 1
  - Hyperglycemia Level 2
- Glucose Level Prediction (mg/dL) (regression)

Further analysis using a confusion matrix shows that the vast majority of samples are correctly classified on the main diagonal, namely:

- 1040 samples in the Normal class were successfully predicted as Normal
- 611 samples of Early Postprandial
- 432 samples of Hyperglycemia Level 1
- 236 samples of Hyperglycemia Level 2.

Misclassification errors are mostly limited and occur primarily between physiologically adjacent classes, such as Low-Normal and Normal, or Hyperglycemia Level 1 and Level 2, which is clinically acceptable. However, caution is advised for errors related to hypoglycemia, as they require further validation.

#### Regression estimation for GlucoNeo check

**FIGURE 3:**
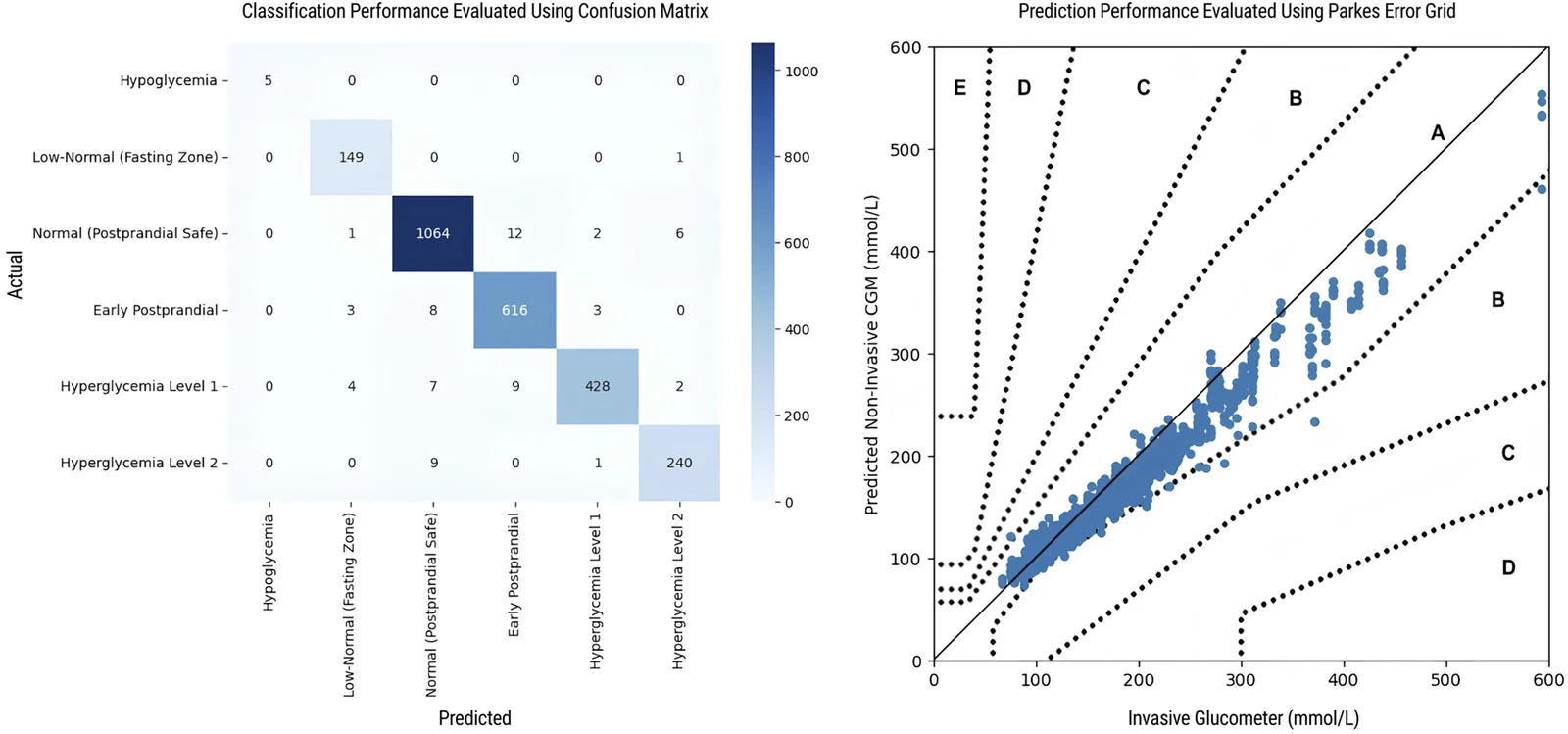
Performance evaluation of the GlucoNeo glucose prediction system. **(A)** Confusion matrix of GlucoNeo Checks classification performance. **(B)** Parkes Error Grid analysis of the Deep Convolutional Neural Network–Long Short-Term Memory–based glucose prediction system (Yogyakarta and Surabaya, Indonesia. 2024–2025).

Clinical validation was conducted to evaluate both the classification performance and clinical accuracy of the proposed DSTNN-based non-invasive CGM model. Figure 3A presents the confusion matrix derived from 2,570 test samples across six glucose categories: Hypoglycemia, Low-Normal (Fasting Zone), Normal (Postprandial Safe), Early Postprandial, Hyperglycemia Level 1, and Hyperglycemia Level 2. The results demonstrate strong diagonal dominance, indicating that the majority of predictions were correctly classified within their respective categories. Notably, the Normal (Postprandial Safe) class achieved the highest number of correct predictions (1,064), followed by Early Postprandial (616), Hyperglycemia Level 1 (428), Hyperglycemia Level 2 (240), and Low-Normal (149), while the Hypoglycemia class recorded 5 correct predictions consistent with its limited sample representation. Misclassifications were minimal and predominantly occurred between adjacent glucose categories for instance, 12 Normal samples were misclassified as Early Postprandial, and 9 Hyperglycemia Level 1 samples were misclassified as Early Postprandial reflecting the natural overlap in physiological glucose transitions rather than systematic model error. Figure 3B presents the Parkes Error Grid analysis, which was employed to assess the clinical significance of the model’s continuous glucose estimation. The system achieved a Mean Absolute Percentage Error (MAPE) of 3.6% ± 0.621, reflecting stable and consistent estimation performance with minimal relative deviation from invasive reference glucometer values. The distribution of all 2,570 data points across the Parkes Error Grid zones reveals that the vast majority of predictions fell within Zone A, indicating clinically accurate estimations that closely agree with reference measurements and would not result in any erroneous clinical action. A small proportion of points were observed in Zone B, primarily at higher glucose concentrations, representing deviations that, while slightly beyond the optimal range, would not lead to dangerous treatment decisions. Critically, no data points were recorded in Zones C, D, or E, which correspond to regions associated with potentially harmful or clinically significant errors. Collectively, these findings confirm that the prediction errors of the proposed model remained well within clinically acceptable limits across the entire glucose measurement range, demonstrating that the device is both technically reliable and clinically safe for deployment in community-based diabetes screening applications.

#### Visualization and implementation in real-time system

Real-time blood glucose monitoring has been enhanced through the development of a dashboard that integrates multi-channel PPG signals with blood glucose data and patient metadata. The system effectively tracks changes in PPG signals while providing stable blood glucose predictions, demonstrating the GlucoNeo Check’s readiness for home healthcare and remote monitoring implementations.

Supplementary Figure 1 illustrates the internet of things-based data acquisition dashboard developed for real-time optical signal monitoring and structured patient metadata recording. The dashboard interface is divided into two.

Each panel is accompanied by a structured User Metadata section that captures essential demographic and anthropometric information, including medical record number, patient initials, age, body weight (kg), height (cm), abdominal circumference (cm), gender, and the corresponding invasive reference glucose level (mg/dL). For the first subject (MUYA, aged 73 years), a reference glucose level of 55 mg/dL was recorded, whereas for the second subject (TRSL, aged 57 years, female), a glucose level of 122 mg/dL was obtained. The dashboard additionally provides JSON and CSV data export options, facilitating downstream data processing and model training pipelines. Overall, this dashboard serves as an integrated platform for synchronized optical signal acquisition and structured clinical metadata management, supporting the development and validation of a non-invasive continuous glucose monitoring system within a community-based diabetes screening setting.

#### Summary of quantitative accuracy

Overall, the study demonstrates that spatiotemporal patterns of PPG signals can be used to predict and classify blood glucose levels through a convolutional and temporal deep learning approach. The integration of IoMT devices, physiological signal processing, and the Deep Convolutional Neural Network–Long Short-Term Memory (CNN–LSTM) model produced a non-invasive glucose monitoring system with a classification accuracy of 89.2% ± 0.0515. GlucoNeo Check shows strong potential as a marketable product and as a reference for developing non-invasive IoMT technology for blood glucose monitoring. This system is expected to improve the quality of life of patients with diabetes and support the P4 medicine paradigm.

The classification system effectively distinguishes between phases and levels of glycemia, minimizing errors mainly in physiologically adjacent classes. This capability supports improved clinical interpretation of continuous glucose prediction results. The diagnostic performance of GlucoNeo Check was further evaluated through a per class analysis based on the confusion matrix results. To address potential concerns regarding class imbalance, we have reported the F1-Score in addition to standard diagnostic metrics as it provides a more balanced evaluation of precision and recall across all glycemic states. The system demonstrated high clinical reliability with sensitivity and specificity exceeding 95% in almost all categories (Table 2). The consistently high Negative Predictive Value (NPV) of over 98.5% across the majority of classes indicates the tool’s effectiveness in ruling out dysglycemia, which is essential for large scale community screening applications.

**TABLE 2:** Per-class diagnostic performance and F1 score of GlucoNeo check (Yogyakarta and Surabaya, Indonesia. 2024–2025).

Glycemic category	Sensitivity (%)	Specificity (%)	PPV (%)	NPV (%)	F1-score (%)	Accuracy (%)
Hypoglycemia	100.00	100.00	100.00	100.00	100.00	100.00
Low-normal (fasting)	99.33	99.67	94.90	99.96	97.06	99.65
Normal (postprandial)	98.06	98.38	97.79	98.58	97.92	98.25
Early postprandial	97.78	98.92	96.70	99.28	97.24	98.64
Hyperglycemia level 1	95.11	99.72	98.62	98.97	96.83	98.91
Hyperglycemia level 2	96.00	99.61	96.39	99.57	96.19	99.26

### Qualitative results

A total of 31 respondents consisting of 20 patients, 8 healthcare professionals, and 3 experts. Collected into one theme, which is the user experience of GlucoNeo Check. The results of document review, in-depth interviews, and focus group discussions (FGD) with respondents involved in using GlucoNeo Check revealed several sub-themes related to user experience. These findings were obtained through source and method triangulation. Source triangulation was conducted by comparing user experiences, healthcare professionals’ perspectives, and cross-disciplinary expert assessments. Method triangulation was then performed using interviews, FGDs, and document review. The resulting problem patterns showed strong thematic consistency, allowing the data to provide strong validation [32].

#### Thematic analysis

Thematic analysis identified user experience as the primary theme in the implementation of GlucoNeo Check, with four interconnected subthemes: device quality and ease of use, comfort of use, simplifying technology, and inclusive interface design (Table 3). Member checking validated these interpretations by reconfirming findings with key informants, ensuring consistency with their experiences. The systematic analysis was documented through an audit trail, covering data collection, coding, theme development, and analytical decisions. Informants emphasized that user experience significantly influences the sustainability, independence, and effectiveness of GlucoNeo Check as a non-invasive blood glucose screening tool in community-based diabetes prevention.

**TABLE 3:** Key themes identified from the qualitative analysis (Yogyakarta and Surabaya, Indonesia. 2024–2025).

No	Theme	Components
1	Perceived device quality and practicality	High-tech device, strong material, practical, independent of needles and alcohol swab
2	Comfort and reduce anxiety in use	Non-invasive, no pain, reduce anxiety, increase safety and confidence for user
3	Technology driven simplicity	Simple user flow, eases users, support monitoring consistency
4	Inclusive interface and ergonomic accessibility	User-friendly across age, less operational obstacle, support widespread use within community

#### Theme 1. Quality and practicality

*“Alat ini canggih ya… saya tidak perlu repot-repot mencari jarum dan kasa alkohol untuk melakukan pemeriksaan glukosa darah.”* (F1)

GlucoNeo Check is a non-invasive digital screening device aimed at enhancing community-based healthcare services. It simplifies blood glucose testing by eliminating the need for auxiliary tools like needles and alcohol swabs. Users have praised its material quality, comfortable design, and attractive appearance, which bolster their confidence in self-screening.

“Saya merasakan bahwa alat ini berasal dari material yang berkualitas, ketika saya pakai sangat nyaman dengan penampilan yang menarik.” (F2)

“Alat ini memiliki kemampuan kerja yang sangat bagus, tanpa tusukan pun saya dengan mudah dapat melakukan pemeriksaan glukosa darah.” (F3)

Respondents assessed that GlucoNeo Check can easily support blood glucose testing with high quality components, allowing users to screen more practically. These findings demonstrate GlucoNeo Check has great potential as a high-quality non-invasive screening tool, ease users and thus supporting primary healthcare and community-based screening sustainably.

#### Theme 2. Comfortability of GlucoNeo check

“Kekhawatiran saya terhadap rasa nyeri saat pemeriksaan glukosa darah terobati dengan kehadiran alat ini.” (F1)

“…saya tidak memiliki rasa cemas ataupun khawatir ketika menggunakan alat ini, sangat praktis dan saya rasa tingkat keamanannya terjamin.” (F2)

“Alat ini sangat cocok untuk masyarakat dengan berbagai kondisi, mudah, gratis, dan memberikan tingkat kenyamanan yang sangat tinggi.” (E4 – Pakar Kesehatan Masyarakat, FGD Pakar)

The GlucoNeo Check device enhances user comfort by providing a non-invasive alternative to traditional blood glucose testing, reducing pain and anxiety. Users report increased confidence and security in self-monitoring, positioning the device as a reliable healthcare tool. This highlights the importance of comfort, particularly regarding pain and anxiety, in ensuring consistent use and supporting self-monitoring sustainability in primary healthcare.

#### Theme 3. Technology that eases users

“Alat ini dirancang dengan teknologi yang berkualitas dengan bantuan sistem AI, untuk mempermudah pasien.” (E5 – Pakar Teknologi Kesehatan, FGD Multidisiplin)

Analysis reveals that GlucoNeo Check is a digital screening device utilizing a data-driven system and AI technology for health monitoring. Experts highlight its quality technology, which simplifies diabetes screening and monitoring for patients. The integration of digital technology and AI offers a strategic advantage for community-based diabetes screening, especially in primary healthcare, where accessible monitoring is essential.

#### Theme 4. Interface design inclusivity

“Desain ini sangat cocok digunakan oleh semua masyarakat, baik muda, dewasa, maupun lansia.” (E6 – Pakar Desain dan Ergonomi Alat Medis, FGD Pakar)

GlucoNeo Check is a modern, community-friendly screening device designed with inclusive principles, ensuring usability across various age groups, including young users. Experts in medical device design confirm its suitability for all societal segments, highlighting its role as an inclusive screening tool. The device’s user-friendly interface is key in enhancing accessibility and promoting sustainable self-screening within communities.

#### Validity of qualitative data

The validity of this qualitative study is upheld through the trustworthiness principle, encompassing credibility, dependability, confirmability, and transferability (Supplementary Figure 2). Credibility is established via method and source triangulation utilizing focus group discussions (FGDs), in depth interviews with patients, healthcare professionals, and experts, and documentation review with member checking to validate findings with key informants. Dependability and confirmability are ensured through a detailed audit trail of all research stages for transparency, while transferability is facilitated by providing clear descriptions of the research context and informant characteristics, allowing readers to evaluate the findings’ applicability to other contexts.

## Discussion

### The accuracy of GlucoNeo check

#### Interpretation of quantitative performance

GlucoNeo Check has great potential as a non-invasive blood glucose monitoring tool, making it suitable for community-based screening and early detection of blood glucose levels in patients. MAPE describes the magnitude of the average relative error between the system’s estimate and the reference value [30]. The reported low MAPE value of 3.6% ± 0.621 suggests high estimation stability; however, this result must be interpreted with caution as such precision may be influenced by the relatively controlled environmental conditions or specific dataset characteristics, suggesting a potential risk of overfitting that requires further external validation. The validation of GlucoNeo Check was conducted through a stepwise and methodologically controlled framework, demonstrating progressive performance maturation from prototype feasibility testing to clinical validation. The system’s predictive accuracy increased substantially from 48.2% at the initial testing stage to 89.2% in Clinical Validation Phase I, indicating improved calibration and stability of glucose estimation under clinically relevant conditions. Importantly, this improvement was accompanied by refinement of clinical interpretability through the expansion of glycemic output categories from 3 to 6 classifications, enabling more granular and actionable screening information for community-based monitoring. To ensure a robust evaluation despite the inherent risk of class imbalance in glycemic datasets, we utilized per-class metrics including sensitivity, specificity, and F1-score. High overall accuracy can often conceal suboptimal performance in minority but clinically critical classes. Specifically, while the system showed high sensitivity for hypoglycemia, we acknowledge that this finding is based on a limited sample size (n = 5) and requires further large-scale validation to avoid dangerous false negatives in real world implementation. Collectively, these findings support GlucoNeo Check as a promising non-invasive screening innovation.

#### Clinical significance

This study uses Clarke Error Grid Analysis as an interpretation approach for the clinical acceptability of glucose estimates, which assesses the extent to which estimation errors can affect clinical decisions. The finding that most of the data falls within Zone A indicates that the majority of estimates are clinically appropriate and do not have the potential to mislead treatment decisions. The presence of a small amount of data in Zone B suggests that there are estimates that are still clinically acceptable but may potentially require caution as they could lead to less than optimal minor decisions. The complete absence of data in Zones C and D indicates that the system did not produce errors likely to lead to clinically dangerous decisions. Nevertheless, the results of this grid still need to be interpreted within the context of the cross sectional design and the reference used, as the Clarke Error Grid is highly influenced by the quality of the reference values and the range of glucose variation represented in the sample.

#### Biological and technical rationale: PPG–glucose–machine learning

Biologically, the PPG signal is affected by changes in peripheral blood volume due to factors such as hemodynamics and vessel elasticity. Blood glucose impacts blood properties and vascular responses, but the relationship with PPG is complex and influenced by temperature, hydration, stress, and activity levels. Machine learning and Convolutional Neural Networks are essential for analyzing the intricate patterns in PPG signal features. The success of the model relies on signal quality and thorough validation to mitigate the risk of overfitting, especially considering that confounding physiological factors were not fully controlled in this field study. Initial findings suggest that the PPG and machine learning approach can function effectively with the GlucoNeo Check, but further validation is necessary to control for these confounding variables.

#### Implications of use in community

Implementing GlucoNeo Check in community settings is highly relevant for preventing and controlling Diabetes Mellitus in Indonesia, where many patients remain undiagnosed. A community-based screening approach is needed to improve early detection rather than relying solely on clinical diagnostic systems. GlucoNeo Check, a non-invasive device using photoplethysmography (PPG), can alleviate the discomfort associated with traditional finger prick methods and extend monitoring capabilities to at risk populations that are often inaccessible through formal healthcare services [33]. The device utilizes machine learning and AI to excel in mass data collection and pattern recognition, leading to reduced human error and time savings in disease prevention [34]. GlucoNeo Check enhances examination adherence in community programs by utilizing non-invasive tools, which reduce pain and anxiety associated with invasive tests. This increase in comfort encourages more frequent blood glucose monitoring, leading to earlier detection and better lifestyle intervention outcomes [35]. Benefits of the device include not only accurate estimation but also its role in promoting consistent monitoring behavior, which is essential for preventing the progression from prediabetes to diabetes [34].

GlucoNeo Check can support community screening by shifting from incidental checks to consistent monitoring of glycemic status. Current screening typically involves one time assessments, hindering the understanding of glucose changes over time. With user friendly, non invasive devices, community based monitoring can be enhanced, facilitating early risk group identification and enabling better referral decisions. Additionally, integrating technology with machine learning and AI can improve users’ comprehension of results, thereby enhancing public health literacy in preventive measures [34]. At the primary healthcare level, GlucoNeo Check enhances resource efficiency by reducing dependency on consumables and expanding coverage. In community settings, tools that are convenient and accessible yield greater impact than highly accurate but infrequent invasive tools. Furthermore, conducting examinations systematically allows monitoring results to aid in population risk mapping and targeted prevention strategies, particularly in high prevalence areas with limited service access [36]. Ultimately, GlucoNeo Check is positioned as a strategic innovation for non-invasive community screening, bolstering a data-driven diabetes prevention ecosystem.

#### Qualitative findings and implementation barriers

The qualitative analysis revealed that while most participants appreciated the comfort and pain free nature of GlucoNeo Check, the reception was not without its challenges. A significant barrier identified among elderly participants was a sense of initial skepticism regarding the validity of a needle free measurement. Some respondents expressed doubt that a device could accurately detect blood glucose levels without direct blood access, often citing their long term familiarity with traditional invasive methods as a point of comparison. Furthermore, technical and operational hurdles were highlighted during the sessions. Participants noted that the device’s high sensitivity to motion artifacts posed a challenge in busy community settings. Specifically, the requirement to remain perfectly still for the duration of the PPG recording was difficult for some elderly users or those with minor tremors, which could lead to failed readings or the need for repeated trials. Additionally, concerns regarding digital literacy emerged, with some participants expressing that they would still require significant assistance from healthcare professionals to operate the device and interpret the results correctly [33, 35]. The identification of these negative experiences is essential for the refinement of community based screening strategies. The skepticism observed suggests that future implementation must be accompanied by targeted health education to build trust in non-invasive technology. Moreover, the feedback regarding motion sensitivity emphasizes the need for further technical optimization in signal filtering to ensure that GlucoNeo Check remains a robust tool for a diverse, real world population.

### Limitations

#### Reference standards

The glucose reference values in this study were obtained using conventional finger prick glucometers rather than the gold standard clinical laboratory plasma glucose analysis. This reliance on capillary blood may introduce noise due to pre-analytical factors such as collection technique, contamination, and hematocrit variations, as well as device specific factors like strip calibration. Consequently, these variabilities may lead to biased model error estimates and limit the overall clinical validity of the findings. Furthermore, the cross sectional nature of this research restricts the ability to evaluate the device’s longitudinal performance and its long term impact on user behavior over time [28].

#### PPG sensitivity to physiological and environmental factors

The PPG signal is highly sensitive and influenced by measurement conditions such as skin and room temperature, movement artifacts, and the user’s peripheral perfusion status [36–38]. Factors affecting blood glucose level estimation include the signal-to-noise ratio and waveform morphology, with physiological and environmental conditions serving as confounding variables. Comprehensive research is required to assess the model’s performance under varied real-world conditions and to establish signal quality indicators for filtering invalid readings [39].

#### Variation in data collection settings

Data collection occurred at various service locations, including Community Health Centers and Hospitals, which enhanced user representation. However, differences in operational characteristics—such as workflow, room conditions, service load, operator training, and patient population—can lead to systematic variations that may impact model performance and generalizability across different settings, potentially decreasing internal validity [39]. Future studies should incorporate location as a covariate, conduct stratified analyses by location, or utilize mixed-effects models to distinguish between-site variation from individual variation.

#### Class imbalance in the classification of glycemic status

The inherent class imbalance within the glycemic dataset poses a risk of biasing aggregate classification metrics toward the majority class [40]. While we addressed this by reporting detailed per class diagnostic performance, we acknowledge that the results for minority but clinically critical classes, such as hypoglycemia, require further large scale validation to mitigate the risk of false negatives. Additionally, the reproducibility of PPG measurements is highly sensitive to physiological and environmental confounders including skin temperature, motion artifacts, and peripheral perfusion status, which were not fully controlled in this field based study. Future developments should employ advanced strategies such as class weighting, re sampling, or focal loss to further enhance model sensitivity across all glycemic ranges.

#### Lack of external validation and limitations in generalizability

Despite promising internal results, the study lacks external validation across diverse ethnic and phenotypic groups, which limits its generalizability due to potential data shift effects on machine learning models [41]. Ideal external validation necessitates independent cross-site testing and variations in device, environmental conditions, and patient profiles. Lacking such validation, GlucoNeo Check’s performance can only be deemed effective for the study’s conditions and population, indicating the need for further validation prior to its clinical adoption as a monitoring standard.

#### Generalizability and environmental factors

Furthermore, the generalizability of these findings is constrained by the specific geographic and environmental contexts of the study, as external validation has not yet been established across diverse populations. It is important to recognize that environmental variables such as ambient temperature and humidity, alongside individual user conditions including peripheral perfusion and skin characteristics, may significantly influence the stability of PPG signals and the resulting blood glucose predictions [37]. Consequently, the current results may not fully represent the performance of GlucoNeo Check in different global populations or under diverse climatic conditions where physiological responses might vary. Future research should prioritize multi center external validation across varied settings to evaluate how these environmental and physiological factors impact device reproducibility and to ensure the robustness of the system for broader international applications.

### Perspective of user’s experience

#### Theme 1: quality and practicality

Non-invasive GlucoNeo Check device was evaluated positively, noted for its user-friendly design and quality, making it beneficial for both healthcare professionals and patients. Users found it easy to learn without needing advanced technical skills. Ease of use correlates with technology acceptance and adoption rates, especially in community healthcare settings, which includes factors like device durability and reading stability, underscoring the importance of optimal protection and efficiency for wearable devices in diverse environments [42]. The quality and ease of use of monitoring tools provide more convenient alternative for blood glucose monitoring, while also improving user comfort [19]. This finding emphasizes the significance of community screening, noting that healthcare workers with heavy workloads may struggle with complex devices, leading to increased procedural errors. Non-invasive blood glucose monitoring tools, like GlucoNeo Check, facilitate easier access to blood glucose data for both healthcare providers and patients, making them practical for daily monitoring [43].

#### Theme 2: comfortability of use

GlucoNeo Check enhances user comfort by alleviating pain associated with traditional invasive blood glucose testing and addressing emotional barriers like anxiety and reluctance. This could lead to improved adherence to testing, which is often hindered by the fear and pain experienced during conventional methods [44]. The fear of pain from using needles in glucose testing heightens pain perception in people with diabetes, leading to reduced willingness to frequently test [36]. These barriers in health behavior can hinder glucose monitoring adherence, reducing early hyperglycemia detection and diabetes management effectiveness. Non-invasive glucose monitoring devices, like GlucoNeo Check, can alleviate emotional distress and fear in diabetic patients, enhancing adherence to monitoring practices and improving their blood glucose conditions, especially in community settings. [45]. This finding emphasizes that non-invasive innovation involves both technology and behavioral interventions. To substantiate claims of improved adherence, further studies should objectively assess adherence through device logs and correlate it with clinical outcomes.

#### Theme 3: technology that eases users

AI integration enhances the interpretation of results, thereby increasing perceived usefulness, particularly in decision support and health literacy by reducing cognitive load. GlucoNeo Check combines machine learning and A.I. to facilitate understanding of blood glucose levels. This non-invasive monitoring technology offers high accuracy, improving comfort and convenience for users, especially diabetic patients. [12]. Integrating AI in healthcare necessitates a focus on transparency and trust, as users and professionals are more likely to accept such systems when they comprehend their fundamental operations, limitations, and the significance of outcomes [46]. A good device should offer minimal explanations sufficient for lay users and provide adequate technical information for healthcare professionals, including uncertainty ranges, signal quality indicators, and warnings for invalid readings.

#### Theme 4: interface design inclusivity

Inclusive interface design is vital in community contexts due to the diversity among target users, including variations in age, reading ability, vision, fine motor skills, and technological experience. Human-centered design principles suggest interfaces should reduce steps, enhance readability, offer clear feedback, and ensure consistency in icons, colors, and text. [47]. GlucoNeo Check features a user-friendly interface that enhances accessibility for people of all ages, particularly benefiting older users who may be less familiar with technology. This design supports community-level self-screening by broadening user reach and reducing cognitive burden [47]. Inclusive UI design is essential for successful adoption among the elderly population, particularly those at high risk for diabetes. A user-friendly interface enhances technology acceptance and improves quality of life for seniors in a digital age [48]. The user interface design of medical wearable devices must be inclusive, taking into account the cognitive and physical abilities of elderly users [49].

### Practical implications

GlucoNeo Check has potential as a community screening device for glucose testing, particularly in high-prevalence, low-awareness areas regarding diabetes. To support scientific claims and readiness for implementation, further research must incorporate plasma glucose references, longitudinal testing, evaluations under various conditions, and validation in diverse populations. Moreover, community-based evaluations should measure the effects on screening adherence, healthcare workflows, and medium-term clinical outcomes to confirm the innovation’s accuracy, impact, and sustainability.

### Conclusion

GlucoNeo Check is a non-invasive blood glucose monitoring device that offers high accuracy suitable for clinical use. Utilizing optical sensors and photoplethysmography (PPG), it eliminates the need for traditional fingerpricks, thus reducing costs and revolutionizing glucometer use. With machine learning and AI capabilities, it enhances data collection and minimizes human error, though further validation is needed for widespread clinical adoption. The device is user-friendly, improving diabetes prevention efforts by alleviating pain concerns of traditional tests, enhancing data collection efficiency, and ensuring accessibility across age groups. It is well-received as an accurate and innovative tool for community-based diabetes prevention.

## Data Availability

The datasets generated and analyzed for this study are not publicly available because they contain potentially sensitive personal information on participants, but are available from the corresponding author upon reasonable request.
